# Phase Transitions of the Majority-Vote Model with Inertia on Directed Erdös–Rényi Networks

**DOI:** 10.3390/e28060591

**Published:** 2026-05-26

**Authors:** Talia Costa Rodrigues, David Santana Alencar, Tayroni Alencar Alves, Gladstone Alencar Alves, Francisco Welington Lima, João Antônio Plascak

**Affiliations:** 1 Dietrich Stauffer Computational Physics Lab, Departamento de Física, Universidade Federal do Piauí, Teresina 64049-550, PI, Brazil; taliadcr@gmail.com (T.C.R.); d.s.m.alencar@gmail.com (D.S.A.); tay@ufpi.edu.br (T.A.A.); 2 Departamento de Física, Universidade Estadual do Piauí, Teresina 64002-150, PI, Brazil; alves.gladstone@gmail.com; 3 Departamento de Física, Centro de Ciências Exatas e da Natureza (CCEN), Universidade Federal da Paraíba, Cidade Universitária, João Pessoa 58051-970, PB, Brazil; 4 Departamento de Física, Universidade Federal de Minas Gerais, C. P. 702, Belo Horizonte 30123-970, MG, Brazil; 5 Centro de Desenvolvimento da Tecnologia Nuclear—CDTN, C. P. 941, Belo Horizonte 30161-970, MG, Brazil; 6 Department of Physics and Astronomy, University of Georgia, Athens, GA 30602, USA

**Keywords:** phase transition, Majoriy Vote model, Erdös–Rényi networks, Monte Carlo simulations, thermodynamic limit, inertia term

## Abstract

The phase transition of the majority vote model with inertia has been investigated by means of extensive Monte Carlo simulations on directed Erdös–Rényi networks. Besides the usual average connectivity and local field that adds the opinion of the site itself, an additional term of inertia is considered. The relaxation time of the average opinion state of the network, together with the average opinion state fourth-order Binder cumulant and the corresponding opinion state susceptibility, have been analyzed for several different network sizes and local field and inertia parameter values, for average connectivity of 20 connections. The present results show that the phase transition of this model strongly depends on the inertia parameter, being quite different and richer than previous results of the same model on other regular networks. For inertia parameters between zero and 0.1 the system undergoes a continuous phase transition; for values in the range 0.1 and 0.2 no transition can be seen; for still larger values of inertia up to 0.5 a first-order phase transition takes place; finally, for values larger than 0.5 the dynamics is fully dominated by the inertia, and again no phase transition is observed.

## 1. Introduction

Models of opinion dynamics with up–down symmetry play a central role in the study of out-of-equilibrium phase transitions [1]. Among them, the majority-vote (MV) model [2] stands out as one of the simplest and most paradigmatic examples of a stochastic system that exhibits a noise-induced order–disorder transition.

In parallel, opinion dynamics has been extensively investigated within the broader framework of sociophysics, a field pioneered by Galam, which applies concepts and methods from statistical physics to describe collective social behavior [3,4,5,6,7]. Subsequent developments have consolidated this approach and highlighted its interdisciplinary relevance [8,9,10].

Within this context, kinetic exchange models provide an alternative and complementary framework for describing opinion formation, where opinions evolve through pairwise interactions among agents [11,12,13,14]. These models display rich nonequilibrium behavior, including spontaneous symmetry breaking and phase transitions [15,16], as well as universality features consistent with the Ising class [17]. More recent extensions have incorporated additional ingredients, such as multistate dynamics and stochastic mechanisms [18,19], further enhancing their ability to capture realistic features of social systems.

These developments complement spin-based approaches such as the majority-vote model, providing a broader perspective on the mechanisms underlying collective behavior and motivating further studies that integrate different modeling ingredients, such as the inclusion of inertia in majority-vote dynamics.

In regular networks, the MV model undergoes a second-order phase transition that belongs to the same universality class as the Ising model [20], as has been established by general symmetry arguments and confirmed by extensive numerical studies. In complex networks, on the other hand, although the up-down symmetry is preserved, the network topology can modify the critical behavior and the exponents associated with the critical transition [21].

Over the years, several generalizations of the majority-vote model have been proposed in order to incorporate more realistic features of social dynamics [22,23,24,25,26,27,28,29,30,31,32]. The extension of the model to random and complex networks revealed that, in the absence of additional mechanisms, the transition remains of second order. Pereira and Moreira [32] have analyzed the MV in Erdös–Rényi (ER) random networks, showing that the critical noise and the critical exponents systematically depend on the average connectivity of the network. Similar results have been achieved by Lima, Sousa and Sumuor [33] in the directed Erdös–Rényi (DER) random networks, in which the direction of connections quantitatively alters the critical point and exponents without modifying the second-order nature of the transition. Here, we have used a directed edge from node *j* to node *i* (It is performed over the in-neighbors of node *i*). As the average connectivity increases, the behavior approaches the mean-field limit, consistent with the general theory for critical phenomena in complex networks [34]. These results consolidated the view that the classical model exhibits a structurally robust and continuous transition.

For many years, however, it was believed that the second-order nature of this transition was robust enough to be insensitive to microscopic changes in dynamics. This perspective began to be revised with the introduction of the concept of behavioral inertia in the MV model. By allowing the probability of a state change of an agent to depend not only on the local majority, but also on its own current state, Chen et al. [35] have demonstrated that, above a critical value of the inertia parameter, the order-disorder transition changes from second-order to a first-order nature. The latter explosive transition is indeed characterized by hysteresis, coexistence of ordered and disordered phases, and exponentially small transition rates with the size of the system. All of these features have been confirmed by Monte Carlo simulations, mean-field theory, and rare-event methods [36]. A notable aspect of this result is its robustness across different complex network topologies, such as Erdős–Rényi networks and scale-free networks.

Subsequent advances have shown that inertia does not need to act homogeneously throughout the system to produce such effects. Harunari, de Oliveira, and Fiore [37] introduced the concept of partial inertia, restricted to a fraction of the vertices, typically those with the highest degree, and demonstrated that discontinuous transitions can be preserved even when only part of the population exhibits inertial behavior. Furthermore, this scenario revealed the emergence of a partially ordered intermediate phase, allowing for the occurrence of two distinct phase transitions. A further refined understanding of the mechanisms responsible for the emergence of these transitions was achieved by Encinas et al. [38], who systematically investigated the fundamental ingredients necessary for the occurrence of discontinuous transitions in the MV model with inertia. The authors showed that low connectivity leads to the suppression of phase coexistence, revealing that not only is inertia a central ingredient for first-order transitions, but also connectivity. Numerical simulations revealed that, for regular random networks, the minimum neighborhood connectivity is 7, while approximately 20 is needed to change the transition order in two-dimensional networks. Therefore, there is a minimum degree of connectivity required for the discontinuity to manifest itself, both in regular random networks and in low-dimensional regular networks.

In this work, we have systematically investigated the order-disorder transition in the majority-vote model with inertia, focusing on the combined role of network topology and average connectivity. We have considered the DER type random networks with a well-defined average connectivity of 20 nodes, as specified by previous works to induce first-order transitions. Through extensive Monte Carlo simulations, we have analyzed the average opinion state of the network, which is analogous to the magnetization in ferromagnetic systems, and its corresponding susceptibility and Binder cumulants. We have also applied finite-size scaling techniques to characterize the order of the transition and the respective critical exponents.

The plan of this paper is as follows. In the next section, the MV model on DER networks is presented, the main quantities of interest are defined, together with the corresponding scaling relations at the second-order phase transition. Some details of the Monte Carlo simulations are also given in Section 2. The results are discussed in Section 3 and some concluding remarks are addressed in the last section.

## 2. Model, Quantities of Interest and Simulations

We have studied a modified version of the original MV model proposed by Chen et al. [35] and defined on DER networks. Thus, besides a local field Θ, which adds the opinion of the site itself, the modification introduced by Chen et al [35] consists of the inclusion of an additional term of inertia θ.

To begin with, let each node *i* of the network (i=1,…,N, where *N* is total number of nodes) be associated with a binary spin variable $\sigma_{i}\in \left\{+1,-1\right\}$. At each time step, a node *i* is randomly selected and tends to align with the local majority of its neighbors, but with a noise probability *q* that allows for misalignment. The single spin-flip probability from $\sigma_{i}$ to $-\sigma_{i}$ is given by

(1) $$w\left(\sigma_{i}\right)=\frac{1}{2}\left[1-\left(1-2q\right)\sigma_{i}S\left(\Theta_{i}\right)\right],$$

where the local field $\Theta_{i}$ is defined as

(2) $$\Theta_{i}=\sum_{j=1}^{k_{i}}\sigma_{j},$$

where the summation is performed over the in-neighbors of node *i* and S(x)=sgn(x) for x≠0, while S(0)=0.

It is clear that in the original MV model above, the state update of a particular node *i* depends exclusively on its neighboring spins. To incorporate extra behavioral inertia, the local field can be modified as

(3) $$\Theta_{i}=\left(1-\theta \right)\sum_{j=1}^{k_{i}}\sigma_{j}/k_{i}+\theta \sigma_{i},$$

where $k_{i}$ is the connectivity degree of the node *i*, that is, the number of connections or neighbors a node has in the network (the in-degree). As a result, the network can also be characterized by an average connectivity 〈k〉. The parameter θ∈[0,0.5] controls the strength of the inertia. For θ=0, the original majority-vote model is recovered. Based on previous simulations [35], for θ≥0.5 the dynamics becomes dominated by inertia, apart from random spin flips with probability *q*, and a spontaneous average opinion state of the network does not emerge. In fact, the fundamental observable of the consensus formation dynamics is the average opinion state of the network, *m*, which measures the level of consensus and is analogous to the magnetization in ferromagnetic systems. It is formally given by

(4) $$m=\left|\sum_{i=1}^{N}\sigma_{i}\left(t\right)\right|/N.$$

When computing the stationary configurations from the simulations, the first $N_{\mathrm{term}}$ Monte Carlo steps have been discarded. The initial configuration corresponds to all node spins in the up (+1) state. Once the system has reached the stationary state, we begin to collect a time series consisting of $N_{t}$ node configurations ${\left\{\sigma_{i}\right\}}_{\ell }$ ($\ell =0,1,\ldots ,N_{t}$) of the system. In order to avoid statistical correlations among system configurations, we also discard 10 Monte Carlo steps between two successive elements of the configuration time series [39]. The interest here lies not only in collecting stationary configurations but also measuring a kind of the relaxation time of the average opinion state. That is why an ordered initial configuration with all opinion states $\sigma_{i}=1$ is considered. From the elements of the time series obtained for each random realization of the network, we can determine the order parameter *M*, the susceptibility χ, and the Binder cumulant *U*, given by the following expressions [35]

(5) $$\begin{array}{ccc} M(q) & = & \left[\langle m\rangle \right],\\ \chi (q) & = & \left[N(\langle m^{2}\rangle -{\langle m\rangle }^{2})\right],\\ U(q) & = & \left[1-\frac{\langle m^{4}\rangle }{3{\langle m^{2}\rangle }^{2}}\right], \end{array}$$

where the symbol 〈…〉 denotes an average taken over the time series, and the symbol $\left[\ldots \right]$ represents the annealed average over the random realizations of the network. All observables are indeed function of the noise parameter *q*.

In this work, the above quantities, as a function of *q*, have been computed through extensive MC simulations on DER networks of finite sizes ranging from N=1800, 3600, 5000, 10,000, and 20,000. In some cases, network sizes of 50,000 and 100,000 have also been simulated. In order to let the system reach its stationary state, the initial $N_{\mathrm{term}}=5\times 10^{5}$ MC steps (MCS) have been discarded. The corresponding time averages have then be computed by taking the next $N_{t}=1\times 10^{6}$ MCS. Here, one MCS consists of randomly choosing *N* sites of the network, as described above. For each set of network size *N* and parameter *q*, $10^{3}$ to $10^{4}$ different configurations have been considered to obtain the configurational averages. For all sets of parameters, we have generated 128 distinct networks.

When one considers a second-order phase transition, the dynamical quantities like in Equation (5) are expected to obey, for very large network sizes, the following finite-size scaling relations

(6) $$\begin{array}{ccc} M & = & N^{-\beta /\nu }f_{M}\left(N^{1/\nu }\left(q-q_{c}\right)\right), \end{array}$$

(7) $$\begin{array}{ccc} \chi & = & N^{\gamma /\nu }f_{\chi }\left(N^{1/\nu }\left(q-q_{c}\right)\right), \end{array}$$

(8) $$\begin{array}{ccc} U & = & f_{U}\left(N^{1/\nu }\left(q-q_{c}\right)\right), \end{array}$$

(9) $$\begin{array}{ccc} q_{max} & = & q_{c}+cN^{-1/\nu }, \end{array}$$

where 1/ν, β/ν, and γ/ν are the critical exponents, similar to the magnetic ones for the corresponding variables, $q_{c}$ is the critical noise and $f_{M,\chi ,U}$ are the respective scaling functions. In the additional last equation, $q_{max}$ has been taken as the noise parameter value locating the maximum of the susceptibility and *c* is a non-universal constant. With a previous knowledge of $q_{c}$, the above equations allow the computation of the desired critical exponents ratio.

For first-order transitions, the exponent ratios in Equations (6)–(9) are simply the dimension of the regular lattice. In the case of networks they should be replaced by the effective dimension of the networks.

## 3. Results

**Figure 1:**
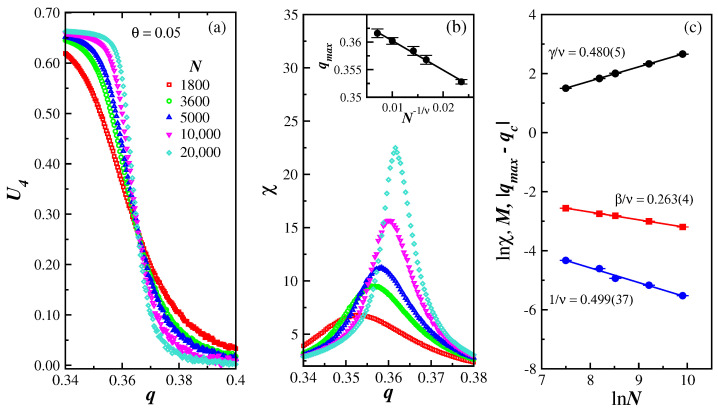
Results of the MV model on DER networks of several sizes *N* with the inertia parameter θ=0.05 and network connectivity 〈k〉=20. The fourth-order Binder cumulant $U_{4}$ is shown in panel (**a**) and the susceptibility in panel (**b**), both as a function of the noise parameter *q*. The logarithm of the maximum value of the susceptibility χ and the order parameter *M* at $q_{c}$, together with the magnitude of $q_{max}$−$q_{c}$, all as a function of the logarithm of the size *N*, are shown in panel (**c**). The legend in (**a**) also applies to (**b**). The intersection point of all sizes in (**a**) indicates a critical noise at $q_{c}$ = 0.3656(2), a value that has been used to obtain the data in panel (**c**). The full lines in (**c**) are linear fits, where the slope is the respective exponent ratio. The inset in (**b**) gives $q_{max}$ as a function of $N^{-1/\nu }$, where the full line is a linear fit with 1/ν obtained from (**c**).

**Figure 2:**
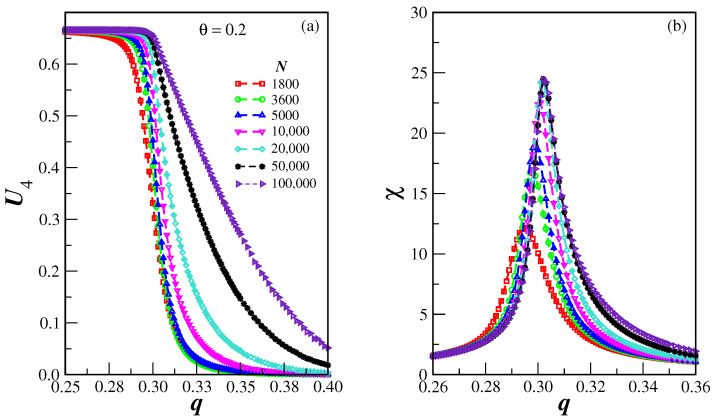
Results of the MV model on DER networks of several sizes *N* with the inertia parameter θ=0.2 and network connectivity 〈k〉=20. The fourth-order Binder cumulant $U_{4}$ is shown in panel (**a**) and the susceptibility in panel (**b**), both as a function of the noise parameter *q*. The legend in (**a**) also applies to (**b**).

**Figure 3:**
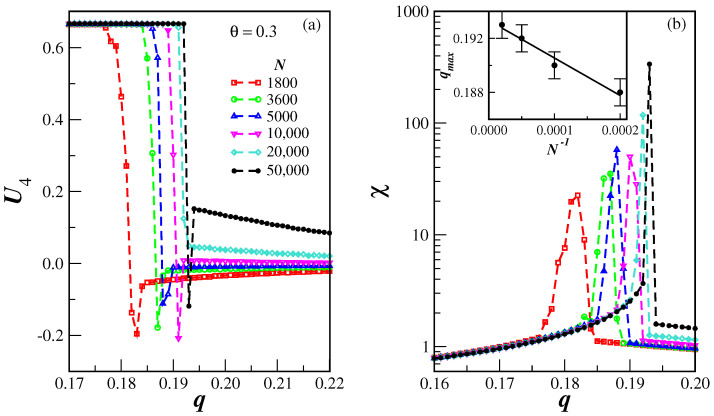
Results of the MV model on DER networks of several sizes *N* with the inertia parameter θ=0.3 and network connectivity 〈k〉=20. The fourth-order Binder cumulant $U_{4}$ is shown in panel (**a**) and the logarithm of the susceptibility in panel (**b**), both as a function of the noise parameter *q*. The legend in (**a**) also applies to the main graph in (**b**). The inset in (**b**) is the linear fit (full line) of $q_{max}$ as a function of $N^{-d_{eff}}$, with $d_{eff}=1$.

Figure 1, Figure 2 and Figure 3 show the fourth-order Binder cumulant [in panels (a)] and the susceptibility [in panels (b)] of the MV model on DER networks for inertia parameters θ=0.05, θ=0.2, and θ=0.3, respectively. In all cases, the average connectivity has been chosen 〈k〉=20.

In Figure 1, for θ=0.05, it can be seen that we have a typical second-order phase transition, as in the case for θ=0. Since the behavior of the order parameter *M* is regular for this transition, only its derivative products have been shown in In Figure 1. There is a noticeable crossing of the fourth-order Binder cumulants in Figure 1a, although they do not intersect at a single point. However, they do cross within a finite interval. When considering lattices $\left(N,N^{\prime }\right)$ with $N^{\prime }<N$, the crossings do not follow a scaling law that allows to determine the critical noise parameter in the thermodynamic limit as usual. Nevertheless, since the crossing interval is quite small, of the order of $10^{-4}$, it allows to consider 1800 < *N* ≤ 20,000 and, from all the data available estimate $q_{c}$ and the corresponding error bar. The result obtained from Figure 1a is *q_c_* = 0.3656(2) with the universal fourth-order cumulant $U_{4}^{*}$ = 0.2676(37).

In Figure 1b the susceptibility is shown for the different network sizes. There is a maximum value of χ, at a certain $q_{max}$ that depends on *N*, that increases as the size increases. With the above $q_{c}$ in hands, it is then possible to compute the order parameter *M* (not shown in Figure 1) and the susceptibility χ, both at $q_{c}$ and resort to the corresponding scaling relations Equations (6) and (7) to estimate the critical exponent ratios β/ν and γ/ν. The log–log plots of the data so obtained are shown in Figure 1c, where the full lines are the linear extrapolations. Equation (9) has also been used to estimate the exponent 1/ν in Figure 1c with $q_{max}$ defined above.

The critical parameters for θ=0.05 are listed in Table 1 in comparison with those without any inertia for θ=0. It can be seen that, within the error bars, the critical exponents with inertia included are the same as those of the model with θ=0, implying the same universality class. With the knowledge of the critical exponent 1/ν we can use again Equation (9) to estimate a new $q_{c}$ from the data already obtained of $q_{max}$. The inset in Figure 1b shows the fit that results in $q_{c}$ = 0.3656(3), in perfect agreement with that coming from the cumulant crossings.

Table 1 also gives the corresponding critical data so obtained for a smaller θ=0.01 and a larger θ=0.09 inertia parameter. It can be seen that, within the error bars, the exponents look the same and the system are in the same universality class as for the original model without any inertia. Moreover, the critical noise parameter seem to have only a small decrease with the inertia parameter, a tendency that will be clearer when considering larger values of the transition noise along the first-order transition.

In Figure 2 for θ=0.2, unlike the results obtained for θ=0.05, we see that the curves of the fourth-order Binder cumulant $U_{4}$, shown in Figure 2a, do not intersect at a specific point $q_{c}$, suggesting no phase transition at non-zero value of *q*. We can also observe in panel Figure 2b that from *N* > 10,000 not only the peaks of susceptibility remain at the same hight as the sizes increase but the entire function tend to collapse for the larger networks. This behavior is a clear indication that there is no phase transition at this value of θ. In fact, this is the case for 0.1 ≤ θ ≤ 0.2. A further indication of no transition for this range of inertia values will be discussed below regarding the behavior of relaxation time of the order parameter as function of the noise parameter.

It is interesting to see the behavior of the fourth-order cumulant and susceptibility for still larger values of the inertia parameter, as it is shown in Figure 3 for θ=0.3. The cumulants in panel (a) have a minimum, typical of a first-order transition, while the susceptibility presents a sharper peak, as is seen by the logarithm plot of χ in panel (b). Indeed, the inset in Figure 3 shows the linear fit of the noise parameter $q_{max}$ of the maximum value of the susceptibility, using the scaling relations Equation (9) with the critical exponent taken as the effective dimension of the network namely $d_{eff}=1$ [33] (on regular lattices the exponent would correspond to the lattice spatial dimension). The extrapolated value in the thermodynamic limit is $q_{c}$ = 0.193(1). Similar results are obtained when considering $q_{max}$ as the minimum of the cumulant in Figure 3a instead (in the scale of those figures, the noise position of some minimum of $U_{4}$ coincide with the maximum of χ). The first-order transition noise value for other inertia parameters are given in Table 1.

**Figure 4:**
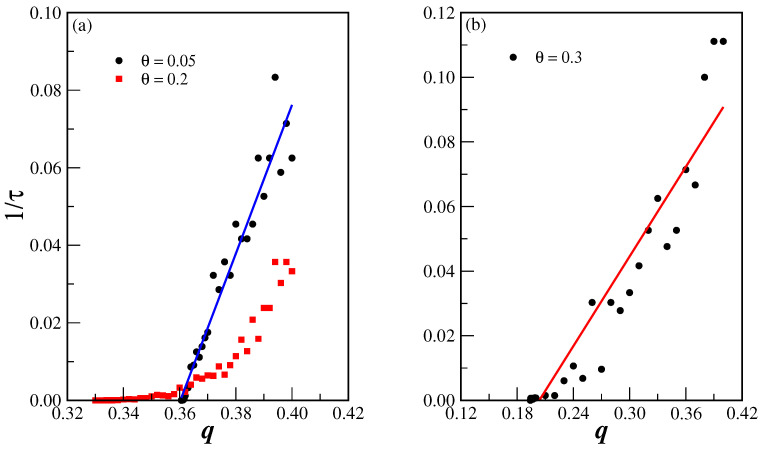
Inverse of the relaxation time τ, as defined in the text, as a function of *q*, for the MV model on DER random networks. Inertia values are θ=0.05 (circles) and θ=0.2 (squares) in (**a**) and θ=0.3 in (**b**). The full lines are linear fits to the data.

There are still two additional ways to better characterize the order of the transitions as the inertia parameter is varied. First, we can look at the behavior of the order parameter as it reaches the stationary regime from the initial state where all opinion states are +1. Defining the value of the Monte Carlo step when the order parameter first becomes negative as a kind of relaxation time τ, as has been done in Refs. [39,40,41], it is possible to have a view of what happens in the initial dynamics when *q* varies. This is shown in Figure 4, where we have the inverse of τ as a function of *q* for 〈k〉=20 and the same values of θ considered so far. Panel (a) considers θ=0.05 and θ=0.2, while panel (b), for questions of clarity, considers only θ=0.3. The full lines are linear fits just to seek the interception of the extrapolation with the noise parameter axis for 1/τ→0, without the meaning of any dynamical exponent because τ is not a true relaxation time. The results in Figure 4a for θ=0.2 give an additional evidence of the absence of a critical transition of the MV model in the DER networks, while for θ=0.05 and θ=0.30 in (b) a finite value for the noise parameter is achieved.

**Figure 5:**
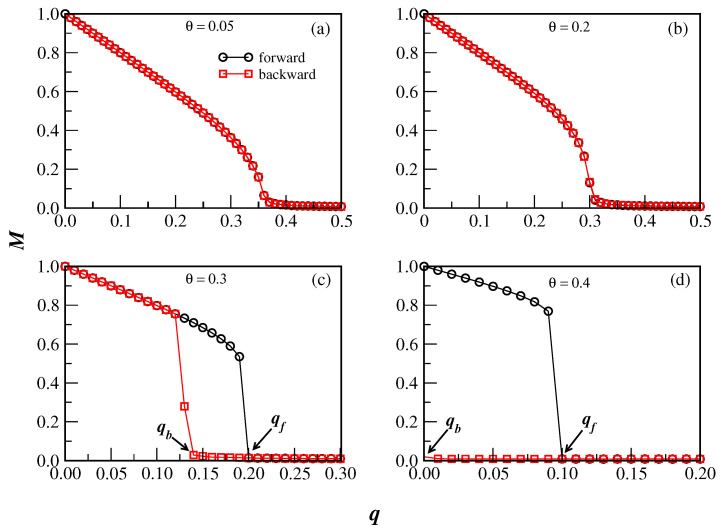
Magnetization *M* as a function of the noise parameter *q* for several values of the inertia parameter θ on DER networks of size *N* = 100,000. Circles are simulations done by increasing *q* from 0 to 0.5 (forward), while squares are simulations for decreasing *q* from 0.5 to 0 (backward). The forward and backward jumps are indicated by $q_{f}$ and $q_{b}$, respectively. The legend in (**a**) also applies to the (**b**–**d**) panels.

Second, we can look at the hysteresis pattern that depicts the dependence of the properties of the system on its past history. Figure 5 shows the absolute value of *M* as a function of *q* for several different values of θ in DER random networks with the size *N* = 10,000 and the average degree 〈k〉=20. The simulation results are obtained by running forward and backward simulations, respectively. The first is done by calculating the steady-state value of *M* as *q* increases from 0 to 0.5 in steps of 0.01. Then, using the final configuration of the last simulation run as the initial condition, runs are performed by decreasing *q* from 0.5 to 0 with the same noise parameter step. For θ=0.05, the forward and backward simulations coincide, implying that we have indeed a second-order transition phase transition, typical of the main feature of the original MV model. For θ=0.2, although there is no phase transition, the forward and backward simulations still match. Interestingly, or θ=0.3 in Figure 5c it can be seen that as *q* increases, the forward magnetization abruptly jumps from nonzero to zero at $q=q_{f}$, with the backward simulations having a jump from zero to a nonzero value at a smaller noise $q=q_{b}$. These two sharp transitions leading to a clear hysteresis loop is characteristic of a first-order transition. By further increasing the value of θ, for example to θ=0.4 in (d), the hysteresis loop becomes wider, despite the decrease in the value of $q_{f}$. Eventually, for θ≥0.4, $q_{b}≃0$.

## 4. Concluding Remarks

The phase transition of the majority vote model with inertia θ has been studied through extensive Monte Carlo simulations on directed Erdös–Rényi random networks with average connectivity 〈k〉=20. Three different regimes have been found as the inertia parameter is varied. For 0≤θ<0.1 the system undergoes a second-order phase transition in the same universality class as the original model without any inertia. For 0.1≤θ≤0.2 the inertial MV model does not exhibit any phase transition for non-zero *q*. For 0.2<θ≤0.5, the inertial MV model undergoes a first-order phase transition. For θ>0.5, the dynamics is fully dominated by the inertia, and no phase transition is observed [35].

**Figure 6:**
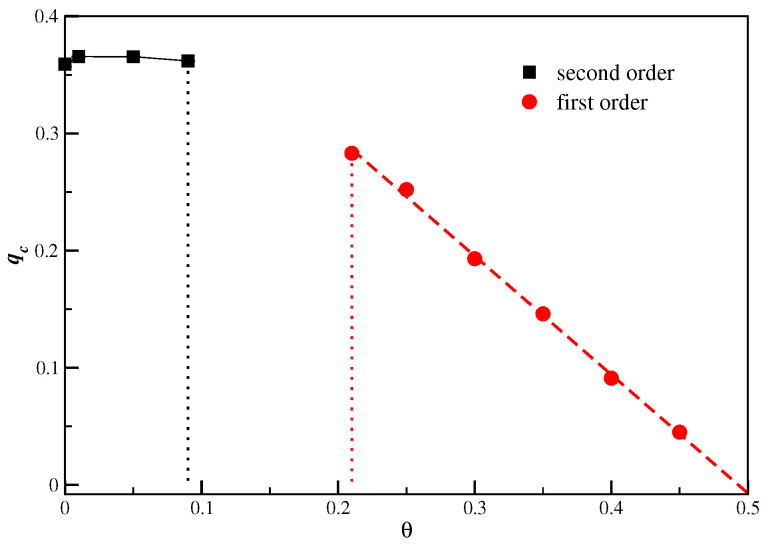
Phase diagram on the inertia versus noise parameters plane of the MV model on DER networks with average connectivity 〈k〉=20. Squares are second-order phase transitions and circles first-order phase transitions. The full line is just guide to the eyes and the dashed line is a linear fit to the first-order transition data. Between the dotted lines there is no transition.

The phase diagram in the inertia versus noise parameters is shown in Figure 6. Apart from the data at θ=0, it seems that there is a tendency of the transition noise value to decrease as the inertia is increased. It should be stressed that the data for θ=0 from Ref. [35] has been obtained with less statistics and considering smaller size networks. The first-order transition line presents a linear decay as

(10) $$q_{c}=0.499\left(7\right)-1.01\left(2\right)\theta ,$$

where $q_{c}$ here generically means the transition noise value. The extrapolation shows that $q_{c}\to 0$ as θ→0.5, as expected (from the linear fit in Figure 6 we have θ = 0.494(8) at $q_{c}=0$).

In addition, there are two interesting features of the phase diagram depicted in Figure 6. Despite the fact that a first-order phase transition actually occurs in this model, there is a lack of transition in the range 0.1≤θ≤0.2 and also a persistent second-order phase transition for small values of θ. These behaviors have not been seen in other models in similar networks (note that, in the present case, the directivity changes the influence of inertia on the phase transition behavior and critical parameters of the system because the undirected case has only continuous and discontinuous transitions [35]). Second, the first-order transition line segment is not bound by two critical points, as in the case of fluids and ordinary magnetic systems.

We have focused here on directed ER networks as a minimal and well-controlled framework, which allows us to isolate the effects of inertia on the collective dynamics without the additional complexity introduced by network heterogeneity or clustering. This choice is consistent with several previous studies on majority-vote dynamics, where ER networks are often used as a baseline to identify fundamental mechanisms. Naturally, extending the analysis to more realistic network topologies is indeed an important direction. In particular, it is well known that scale-free networks, such as Barabási–Albert networks, introduce strong degree of heterogeneity, which can significantly affect critical behavior. Likewise, small-world networks with high clustering may alter the balance between local consensus formation and long-range interactions. In this context, the presence of hubs in scale-free networks is expected to enhance the stability of ordered states; clustering may favor local consensus and potentially modify the nature of phase transitions; and directed structures combined with heterogeneity could further enrich the dynamical behavior. Nevertheless, a systematic investigation on more realistic networks, as well as different values of the average degree 〈k〉, is left as an interesting direction for future works.

## Figures and Tables

**Table 1:** Critical values of the majority-vote model on DER networks with average connectivity 〈k〉=20 for several values of the inertia parameter θ. For inertia θ≥0.21 a first-order transition takes place.

θ	$q_{c}$	β/ν	γ/ν	1/ν	$U_{4}^{*}$
0 *	0.359(2)	0.280(4)	0.485(2)	0.510(10)	0.258(4)
0.01	0.3656(4)	0.261(7)	0.488(4)	0.447(69)	0.272(8)
0.05	0.3656(2)	0.263(8)	0.480(6)	0.499(37)	0.268(4)
0.09	0.3618(2)	0.253(5)	0.492(4)	0.52(2)	0.268(4)
0.21	0.283(2)	–	–	–	–
0.25	0.252(1)	–	–	–	–
0.3	0.193(1)	–	–	–	–
0.35	0.146(1)	–	–	–	–
0.4	0.091(1)	–	–	–	–
0.45	0.045(2)	–	–	–	–

* From reference [33].

## Data Availability

The original contributions presented in this study are included in the article. Further inquiries can be directed to the corresponding authors.

## Abbreviations

The following abbreviations are used in this manuscript:

MV	majority-vote
ER	Erdös–Rényi
DER	directed Erdös–Rényi

## References

[1] Castellano C., Fortunato S., Loreto V. (2009). Statistical physics of social dynamics. Rev. Mod. Phys..

[2] de Oliveira M.J. (1992). Isotropic majority-vote model on a square lattice. J. Stat. Phys..

[3] Galam S. (2016). Sociophysics: A Physicist’s Modeling of Psycho-Political Phenomena.

[4] Galam S., Gefen Y., Shapir Y. (1982). Sociophysics: A new approach of sociological collective behaviour. J. Math. Sociol..

[5] Galam S. (1997). Rational group decision making: A random field Ising model at *T*=0. Phys. A.

[6] Galam S. (2002). Minority opinion spreading in random geometry. Eur. Phys. J. B.

[7] Galam S. (2008). Sociophysics: A review of Galam models. Int. J. Mod. Phys. C.

[8] Sen P., Chakrabarti B.K. (2014). Sociophysics: An Introduction.

[9] Perc M. (2019). The social physics collective. Sci. Rep..

[10] Jusup M., Holme P., Kanazawa K., Takayasu M., Romić I., Wang Z., Perc M. (2022). Social physics. Phys. Rep..

[11] Toscani G., Sen P., Biswas S. (2022). Kinetic exchange models of societies and economies. Phil. Trans. R. Soc. A.

[12] Lallouache M., Chakrabarti A.S., Chakraborti A., Chakrabarti B.K. (2010). Opinion formation in kinetic exchange models: Spontaneous symmetry-breaking transition. Phys. Rev. E.

[13] Sen P. (2011). Phase transitions in a two-parameter model of opinion dynamics with random kinetic exchanges. Phys. Rev. E.

[14] Biswas S. (2011). Mean-field solutions of kinetic-exchange opinion models. Phys. Rev. E.

[15] Biswas S., Chatterjee A., Sen P. (2012). Disorder-induced phase transition in kinetic models of opinion dynamics. Phys. A.

[16] Mukherjee S., Chatterjee A. (2016). Disorder-induced phase transition in an opinion dynamics model: Results in two and three dimensions. Phys. Rev. E.

[17] Mukherjee S., Biswas S., Chatterjee A., Chakrabarti B.K. (2021). The Ising universality class of kinetic exchange models of opinion dynamics. Phys. A.

[18] Biswas S., Chatterjee A., Sen P., Mukherjee S., Chakrabarti B.K. (2023). Social dynamics through kinetic exchange: The BChS model. Front. Phys..

[19] Biswas K., Sen P. (2022). Nonequilibrium dynamics in a three-state opinion-formation model with stochastic extreme switches. Phys. Rev. E.

[20] Grinstein G., Jayaprakash C., He Y. (1985). Statistical mechanics of probabilistic cellular automata. Phys. Rev. Lett..

[21] Dorogovtsev S.N., Goltsev A.V., Mendes J.F.F. (2008). Critical phenomena in complex networks. Rev. Mod. Phys..

[22] Lima F.W.S. (2012). Three-state majority-vote model on square lattice. Phys. A.

[23] Vilela A.L.M., Moreira F.G.B. (2009). Majority-vote model with heterogeneous agents. Phys. A.

[24] Vilela A.L.M., Stanley H.E. (2018). Effect of strong opinions on the dynamics of the majority-vote model. Sci. Rep..

[25] Choi J., Goh K.-I. (2019). Majority-vote dynamics on multiplex networks with two layers. New J. Phys..

[26] Vilela A.L.M., Zubillaga B.J., Wang C., Wang M., Du R., Stanley H.E. (2020). Three-state majority-vote model on scale-free networks and the unitary relation for critical exponents. Sci. Rep..

[27] Kim M., Yook S.-H. (2021). Majority-vote model with degree-weighted influence on complex networks. Phys. Rev. E.

[28] Vilela A.L.M., Pereira L.F.C., Dias L., Stanley H.E., da Silva L.R. (2021). Majority-vote model with limited visibility: An investigation into filter bubbles. Phys. A.

[29] Lima J.R.S., Lima F.W.S., Alves T.F.A., Alves G.A., Macedo-Filho A. (2022). Diffusive majority-vote model. Phys. Rev. E.

[30] Zubillaga B.J., Vilela A.L.M., Wang M., Du R., Dong G., Stanley H.E. (2022). Three-state majority-vote model on small-world networks. Sci. Rep..

[31] Ryu S., Kwak W. (2024). Critical behavior of three-state majority-vote model on Kagomé lattice. New Phys. Sae Mulli.

[32] Pereira L.F., Moreira F.G.B. (2005). Majority-vote model on random graphs. Phys. Rev. E.

[33] Lima F.W.S., Sousa A.O., Sumuor M.A. (2008). Majority-vote model on directed Erdos–Rényi random graphs. Phys. A.

[34] Chen H., Shen C., He G., Zhang H., Hou Z. (2015). Critical noise of majority-vote model on complex networks. Phys. Rev. E.

[35] Chen H., Shen C., Zhang H., Li G., Hou Z., Kurths J. (2017). First-order phase transition in a majority-vote model with inertia. Phys. Rev. E.

[36] Chen H., Shen C., Zhang H., Kurths J. (2017). Large deviation induced phase switch in an inertial majority-vote model. Chaos.

[37] Harunari P.E., de Oliveira M.M., Fiore C.E. (2017). Partial inertia induces additional phase transition in the majority vote model. Phys. Rev. E.

[38] Encinas J.M., Harunari P.E., de Oliveira M.M., Fiore C.E. (2018). Fundamental ingredients for discontinuous phase transitions in the inertial majority vote model. Sci. Rep..

[39] Rodrigues T.C., Alencar D.S., Alves T.A., Alves G.A., Lima F.W., Plascak J.A. (2026). Absence of phase transition in the Biswas–Chatterjee–Sen model on directed Barabási–Albert networks. Symmetry.

[40] Sumour M.A., Shabat M.M. (2005). Monte Carlo simulation of Ising model on directed Barabási–Albert networks. Int. J. Mod. Phys. C.

[41] Sumour M.A., Shabat M.M., Stauffer D. (2006). Absence of ferromagnetism in Ising model on directed Barabási–Albert networks. Int. J. Mod. Phys. C.

